# Customized Metal 3D Printed Total Wrist Prosthesis in the Treatment of Severely Destroyed Wrist: Design Rationale and Clinical Applications

**DOI:** 10.1111/os.70030

**Published:** 2025-03-26

**Authors:** Qipei Wei, Chang Liu, Shanlin Chen, Min Liu

**Affiliations:** ^1^ Department of Hand Surgery Peking University Fourth School of Clinical Medicine Beijing China; ^2^ Department of Hand Surgery Beijing Jishuitan Hospital, Capital Medical University Beijing China; ^3^ Beijing Research Institute of Traumatology and Orthopaedics Beijing China; ^4^ AK Medical Beijing China

**Keywords:** 3D‐print, bone defect, custom‐made, prosthesis, wrist

## Abstract

**Objective:**

Severe destruction of the wrist joint after trauma, disease, or iatrogenic causes can lead to significant bone defects and deformities, limiting the options for surgeries. Bespoke 3D‐printed metal prostheses were designed and used for four patients. This study aimed to describe the design rationale and report the clinical outcomes.

**Methods:**

Between 2022 and August 2024, we followed up on four patients with significant bone defects and deformities caused by various factors, who opted against arthrodesis. These patients were treated with customized 3D‐printed prostheses replacements. All cases underwent assessment through clinical and radiological examinations, evaluating parameters including passive range of motion (ROM) of the wrist, grip strength, VAS of pain, and functional scales such as the Mayo score, Patient‐Rated Wrist Evaluation (PRWE), and Quick Disabilities of the Arm, Shoulder, and Hand (quick DASH) score. A paired *t*‐test was conducted to compare pre‐ and postoperative outcomes.

**Result:**

The mean follow‐up period lasted 11.9 ± 6.7 months (range 6–18 months). All patients reported satisfying pain relief and enhanced function. The average ROM was 23.3° ± 5.7° of extension and 27.0° ± 18.6° of flexion. The average VAS score of pain was 1.5. The mean Mayo score, PRWE, and Quick DASH were 60.0, 31.5, and 28.3, respectively. No complications such as loosening, dislocation, or infection were observed during the follow‐up period.

**Conclusions:**

Customized 3D‐printed prosthetic replacements for severely destroyed wrists can provide good functional outcomes with a higher satisfaction rate.

**Level of Evidence:**

IV.

## Graphical abstract

Four patients with severe wrist destruction from various causes were treated with customized 3D‐printed prostheses. In addition to the specific articular surface matching their original radiocarpal joint, two innovative proximal component designs were created to address different bone defects. Good functional outcomes and high satisfaction rates were achieved.

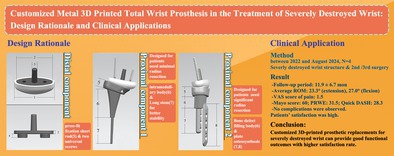

## 1 Introduction

Since their development in the 1970s, total wrist prostheses have undergone four generations of refinement. As an alternative to arthrodesis, the advantage of total wrist arthroplasty (TWA) lies in preserving range of motion (ROM) while providing similarly effective pain relief. Today, TWA has demonstrated fairly acceptable outcomes in treating conventional indications such as rheumatoid arthritis, SLAC/SNAC, and osteoarthritis [1, 2]. However, in cases of severely compromised bone structure due to reasons like severe trauma, tumors, or failed reconstructive surgery, TWA is still rarely chosen.

For severely damaged wrist joints, the primary challenge is the extensive bone defects resulting from the lesion resection and the significant bone deformation. The surgical options are limited. Wrist arthrodesis, while providing satisfactory pain relief and a lower complication rate, results in complete loss of joint mobility [3]. Reconstruction with vascularized or nonvascularized autografts, as well as osteoarticular allografts, can offer better function but comes with a relatively high rate of complications and failure [4, 5]. Reports on using prostheses to treat large bone defects posttumor excision have shown promising outcomes [6, 7, 8, 9]. However, the majority of these studies focused on hemi‐wrist prosthetic designs, which may not be suitable for patients with extensive articular destruction. Customized total wrist prosthesis replacements following en‐bloc resection have been performed in several cases, with satisfactory outcomes observed over a 10‐year follow‐up period [10, 11]. The customized prostheses enable more flexible and biomechanically suitable individual designs, demonstrating excellent stability and durability. However, studies exploring their effectiveness for wrists with severe joint damage and bone defects remain scarce. We hypothesize that total wrist replacement using custom 3D‐printed metal prostheses could be a viable option for treating these complicated conditions.

In this study, our purpose is: (i) report a case series of severely damaged wrists treated with customized 3D‐printed total wrist joint prostheses; (ii) share the design rationale behind the prostheses; (iii) evaluate the functional outcomes during medium‐term follow‐up.

## 2 Materials and Methods

### 2.1 Patients

This retrospective study utilized data from four patients treated with customized mental 3D‐printed total wrist prostheses between 2022 and August 2024. The study was approved by the Ethics Committee of Jishuitan Hospital, under approval number K2024‐135‐00, and all procedures were conducted in accordance with ethical standards and regulations. All patients provided written informed consent to participate in this study.

The inclusion criteria were as follows: (1) wrist joint structural destruction due to various causes (trauma, tumors, iatrogenic, etc.) that cannot be repaired with autologous bone graft or standardized wrist arthroplasty methods; (2) severe limitation of wrist function (flexion‐extension arc ≤ 30°, or VAS ≥ 4 for 6 months, or PRWE ≥ 50, or Quick DASH ≥ 60); (3) refusal to undergo arthrodesis; (4) age > 18 years; (5) follow‐up time > 6 months. The exclusion criteria were as follows: (1) active infection or osteomyelitis; (2) severe osteoporosis (*t*‐score ≤ −3.0); (3) wrist extensor/flexor muscle strength < 3, or irreversible damage to the forearm neurovascular bundle, or joint stiffness due to scar tissue or other pathological factors affecting wrist function; (4) inability to comply with rehabilitation or attend regular follow‐up visits.

Among the four included patients, two were initially diagnosed with giant cell tumors (GCTs) of the radius, while the other two suffered from severe trauma as the primary cause. All four patients had undergone primary or multiple surgeries, which resulted in rather unsatisfactory outcomes. This prompted them to seek further improvement.

**FIGURE 1:**
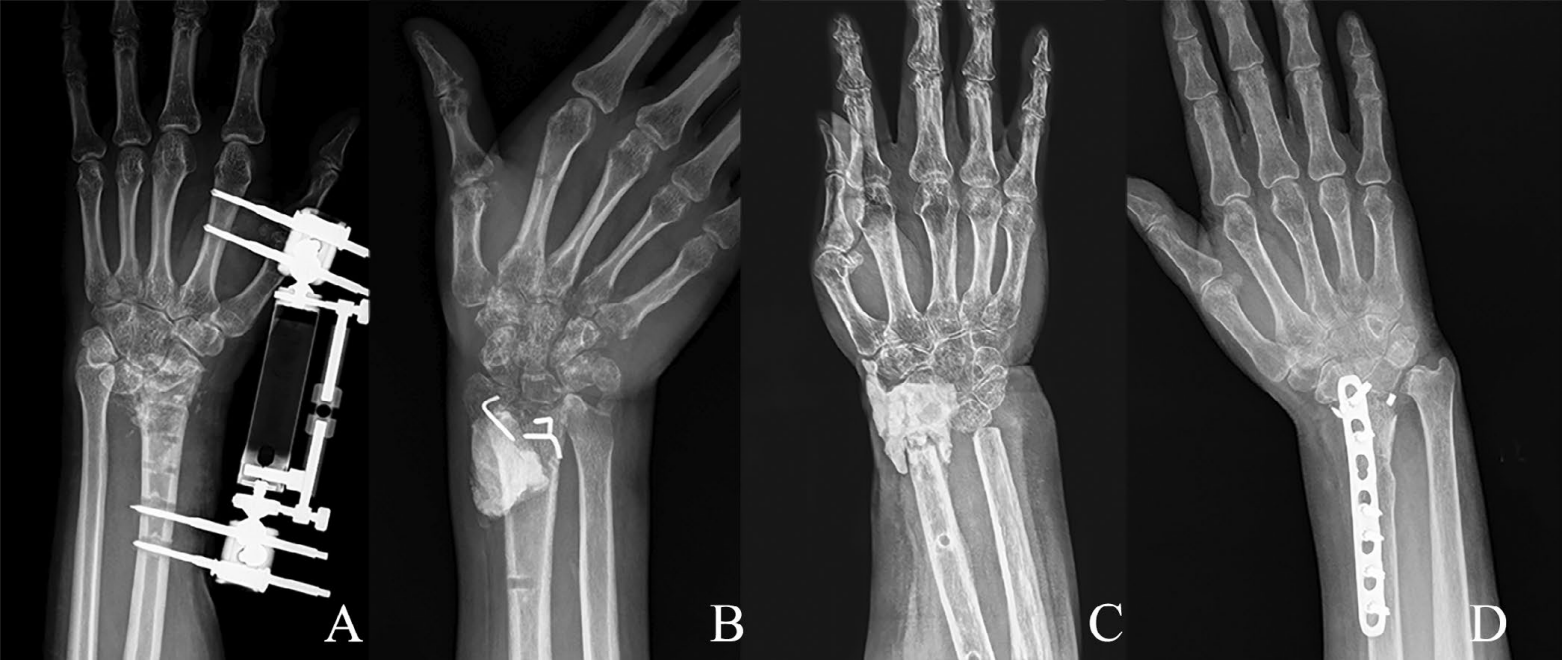
Preoperative radiographies of patients. (A) Patient 1: 3 months after plate removal and the external fixator lengthening procedure. (B) Patient 2: 1 month after the removal of the Kirschner wires and bone cement. Narrowing of the radiocarpal space, destruction of the articular surface, and osteoporosis of the carpal bones are observed. (C) Patient 3: 6 months after cement filling. (D) Patient 4: 3 years after the excision of a GCT and allograft reconstruction.

Patient 1 was diagnosed with a GCT of the distal radius. The patient was treated with en‐bloc resection followed by iliac bone graft reconstruction. Sixteen months postoperatively, the patient gradually experienced restricted movement and significant wrist dislocation, which was managed with internal fixation. Sixteen months after the initial operation, the plate was removed, and external fixator lengthening was performed (Figure 1A).

Patient 2 suffered a comminuted open fracture of the right wrist due to a mountain bike accident. Emergency treatment involved debridement and bone cement reconstruction of the radius bone defect. After the removal of the bone cement, a significant deformity developed. Preoperative radiography showed osteoporosis in the carpal bones (Figure 1B).

Patient 3's wrist was severed due to a cutting injury. After replantation surgery, residual forearm shortening and defects in the ulna and radius persisted. Seven months later, the external fixation was removed, and cement was implanted to restore wrist height. Six months after that, the patient visited the clinic seeking ways to improve wrist function (Figure 1C).

Patient 4 underwent excision of a GCT of the radius and allograft reconstruction at another hospital 3 years earlier (Figure 1D). The patient experienced chronic pain and limited mobility postoperatively. Three months before the TWA, due to poor healing and joint collapse at the distal radius, the patient underwent joint debridement and external fixation, resulting in a significant bone defect.

The average age of the patients was 39.5 ± 14.6 years (range 26–57 years), with three involving the right wrist and one involving the left wrist. Some of the patients (1 and 2) were relatively young for the conventional indications for TWA. However, both of them refused to accept arthrodesis despite being informed of the associated risks for TWA. Preoperative data collected for all patients included basic demographic information, wrist ROM, grip strength, Visual Analog Scale (VAS) score of pain, and three hand function scores: Patient‐Rated Wrist Evaluation (PRWE), Quick Disabilities of the Arm, Shoulder, and Hand (Quick DASH) score, and Mayo score. Additionally, wrist X‐rays and computed tomography (CT) scans were performed.

### 2.2 Prostheses Design Rational

All four patients received customized mental 3D‐printed total wrist prostheses for replacement. The prosthesis design was based on CT data from the contralateral wrist, which has a normal anatomical bone structure.

**FIGURE 2:**
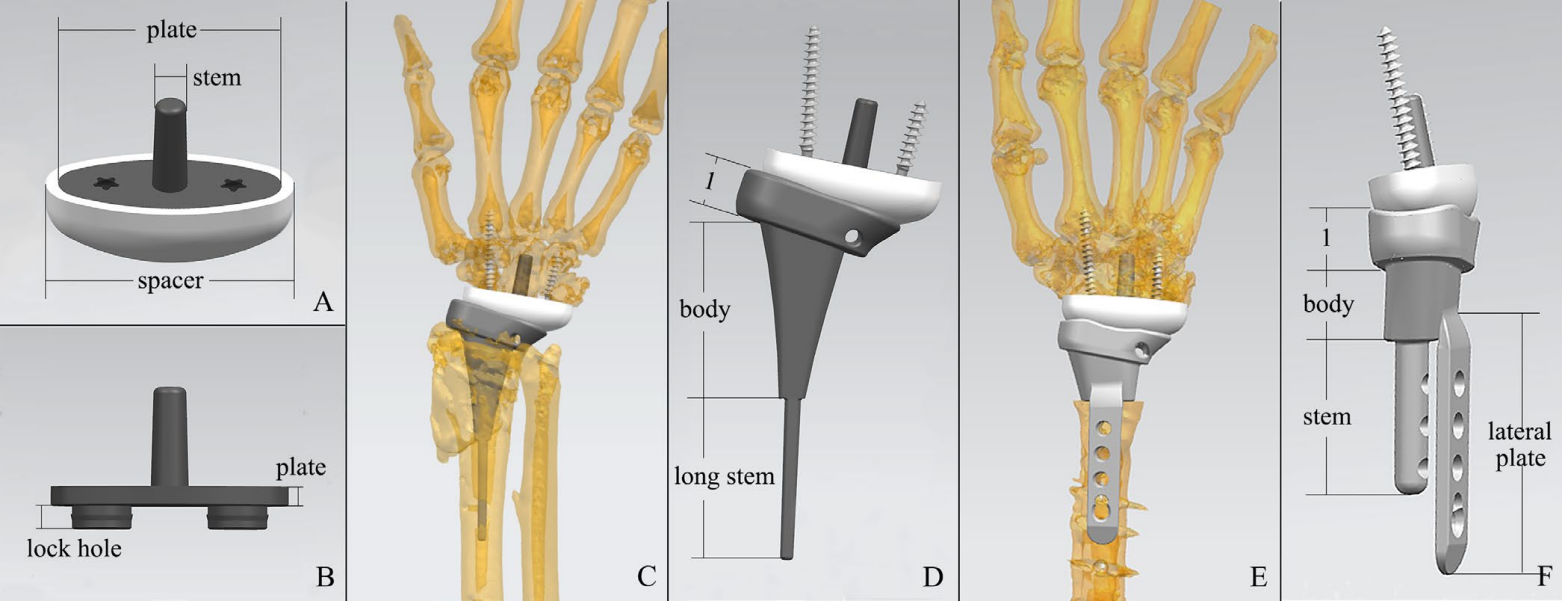
Appearance of the customized 3D‐printed prosthesis. (A, B) The Appearance of the distal component. The design comprises four main components: A spacer with an articular surface contoured to fit the carpal side of the radiocarpal joint, a titanium alloy plate providing support for the spacer, a fixation stem integrated into the plate, and two universal screws for additional stabilization. (C, D) The appearance of the prosthesis design for the patient with minimal bone resection. Three components: The articular surface structure (D1), a body designed to fill the remaining medullary cavity, and a long stem measuring approximately 1–1.5 times the body's length. (E, F) The appearance of the prosthesis design for the patient underwent extensive bone resection. (F1) The articular surface structure. The body was designed to match the contour of the contralateral radius. For added stability, a plate extending from the edge of the body was included. The intramedullary stem features holes aligned with those on the plate to accommodate through‐screws.

The prosthesis comprises a proximal component and a distal component. The proximal component was integrally printed using cobalt‐chromium‐molybdenum alloy and comprises three parts: the articular surface structure, the body, and the fixation stem. The distal component consists of four parts: an ultra‐high molecular weight polyethylene (UHMWPE) spacer, whose articular surface shape fits the carpal side of the radiocarpal joint; a titanium alloy plate supporting the spacer; a fixation stem on the plate; and two universal screws for additional fixation (Figure 2A,B). The fixation rod is designed with a 3D‐printed porous alloy structure for press‐fit fixation in the capitate. The radial screw is precisely long enough to pass through the base of the second metacarpal, while the other screw extends toward the ulnar side without entering the fourth CMC joint. The articular surface structure of the proximal component has a concave surface that fits the distal articular surface but is slightly larger to allow for some sliding. For patients with significant bone resection, a hole should be left on the ulnar side of the articular surface structure to facilitate tendon suturing.

Depending on how much the distal radius bone is preserved, two alternative designs for the body and stem of the proximal component have been developed (Figure 2C–F). For patients requiring minimal radius resection but with malunited bone structures at the distal radius (e.g., Patient 2), the body was created based on the remaining medullary cavity to fill the deformed area. To promote bone tissue ingrowth, the outer surface of this alternative was coated with a 3D‐printed porous alloy structure. For patients requiring significant resection of the distal radius, the body was constructed to match the contour of the contralateral radius.

To achieve a more robust fixation, two alternative fixation designs were developed to complement the body designs. The first method involves a long stem for the intramedullary body design (Figure 2D). The length, based on the long‐stem prostheses used in knee and hip surgeries, was set to approximately 1–1.5 times the length of the body [12, 13, 14]. This design is primarily based on considerations of the bone quality in the distal radius. In the two patients we encountered who did not require extensive bone resection, both had significant osteoporosis in the distal radius due to previous severe arthritis. The long stem design helps transfer more lateral stress to the proximally stronger cortical bone, thereby minimizing the risk of potential periprosthetic fractures. In fact, for patients with recurrent periprosthetic fractures caused by severe osteoporosis, the use of long‐stem prostheses is a common and effective strategy [15, 16].

The second method employed a combination of intermedullary stem and plate osteosynthesis for the second body design in patients who underwent extensive bone resection (Figure 2F). In addition to the regular stem, a plate extends from the proximal edge of the body. The intramedullary stem has holes aligned with the plate holes to accommodate the through screws. For patients requiring substantial osteotomy, the space created by resection is filled with the heavy cobalt‐chromium‐molybdenum alloy body of the proximal component. Therefore, the area directly exposed to compression and traction forces from surrounding soft tissues is increased. As a result, preventing loosening caused by the body becomes a critical concern. To address this, we incorporated a lateral plate directly connected to the body to provide extra resistance to axial traction and lateral forces [12, 17]. Additionally, we placed a 3D‐printed porous alloy structure at the interface between the implant body and the cortical bone. Furthermore, no long rods were used in this design, as the severely osteoporotic section was removed along with the distal radius in the two patients using this design.

### 2.3 Surgical Technique and Postoperative Management

**FIGURE 3:**
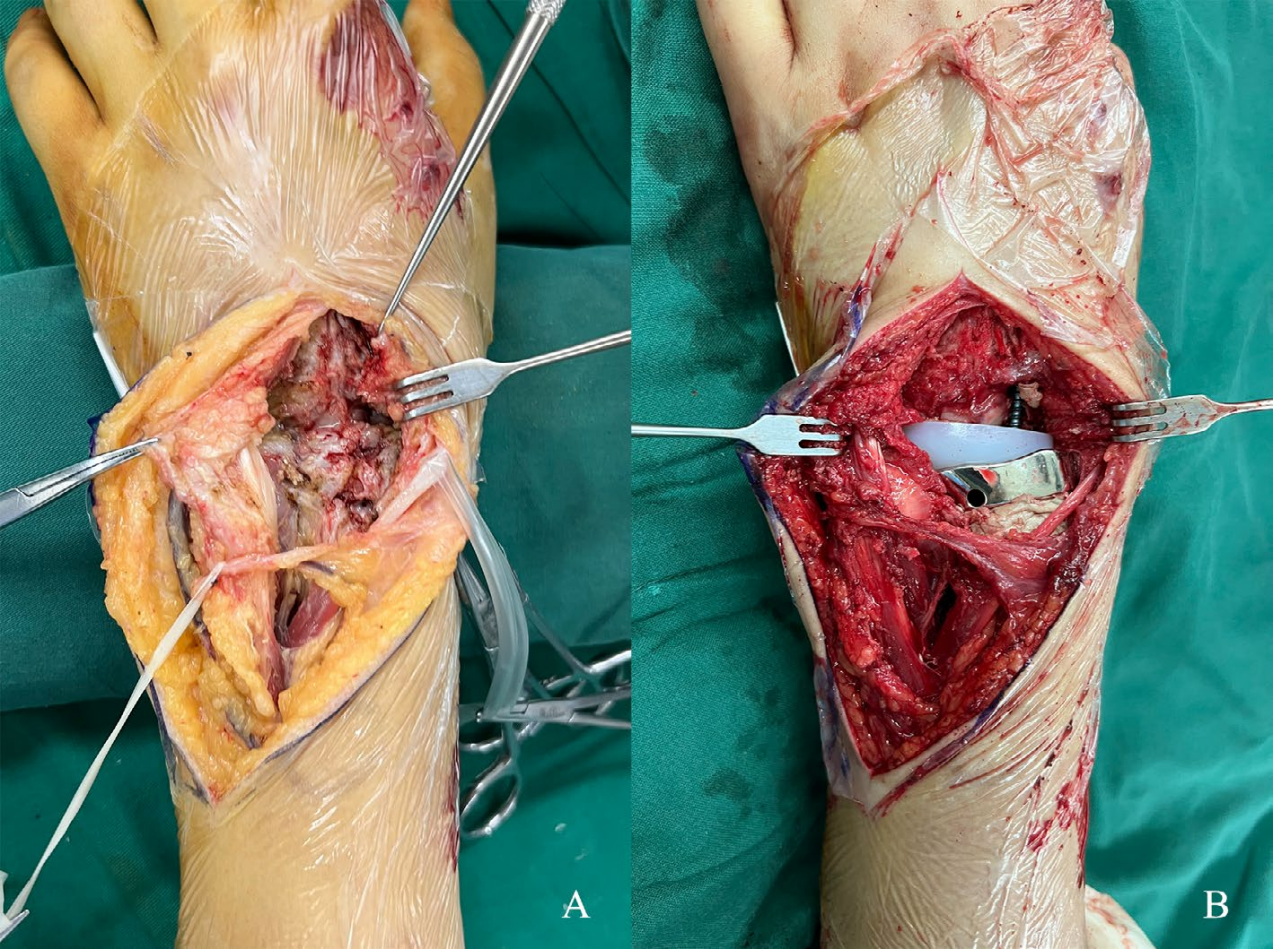
Surgical procedure of Patient 1: (A) A longitudinal dorsal approach was used to open the joint capsule, revealing severe joint destruction with cartilage defects at the distal radius and proximal carpal bones, along with carpal displacement. The scaphoid, lunate, triquetrum, and the proximal articular surface of the capitate were removed during the surgery. The residual articular surface of the distal radius and hardened bone tissue were excised. (B) The appearance following the insertion and fixation of the prosthesis.

The patients were operated on under brachial plexus anesthesia. The affected upper limb was prepped and draped, with a tourniquet pressure set to 260 mmHg. A standard dorsal approach was made to the wrist joint (Figure 3A). Osteotomies of the proximal carpal bones and the proximal articular surface of the capitate were performed. According to the condition of the patient, a specific length of the distal radius was resected. Care was taken to protect surrounding ligaments, tendons, soft tissue, and the joint capsule. The medullary cavity of the radius was reamed, and a trial of the appropriately sized customized proximal component was performed. For the carpal side, a guide pin was inserted into the proximal capitate, and imaging confirmed proper placement before reaming and trialing the component. The trial implants were assembled to ensure that the tension of the soft tissues was acceptable. Once the size and placement of the prosthesis were confirmed, the distal component was implanted first and secured with two cancellous bone screws. After inserting the proximal component into the radius, the ligament was sutured into the hole on the proximal articular surface structure, if necessary. The joint was then reduced, and stability and ROM were checked again (Figure 3B). Hemostasis was carefully achieved before wound closure, and a drain was placed, which was removed on the first postoperative day.

For patients who experience pain and limitations in supination‐pronation movements, a wafer procedure will be performed in conjunction with the resection of the distal radius.

Postoperatively, patients wore a brace to immobilize the wrist in a neutral position. The brace was removed after 3 weeks to begin mild functional exercises, with intensity gradually increased based on the patients' tolerance. All patients were advised to avoid heavy labor and excessive stress on the wrist.

### 2.4 Clinical Evaluation and Follow‐Up

Intraoperative indicators, such as blood loss and operative time, were recorded. All patients were invited to participate in follow‐up and signed informed consent forms before surgery. Clinical follow‐ups were scheduled at 6 weeks, 3 months, 6 months, 1 year, and annually thereafter. All patients returned to the hospital for follow‐up physical and imaging examinations.

Clinical and X‐ray examinations were performed on the patients. The ROM of the forearm and wrist was measured with a goniometer. Grip strength and pinch strength were measured using a dynamometer and a pinch gauge, respectively. Subjective reporting of pain was done using the VAS of pain. Other subjective functional scores included QuickDASH, PRWE, and Mayo wrist scores. All patients were asked two additional questions: What are your expectations for your operated wrist in the future? If given the opportunity to choose again, would you undergo this operation? All patients were followed up at their final visit by a staff member who was blinded to the surgical procedure.

### 2.5 Statistic Methods

All statistical analyses were conducted using SPSS (version 27.0, IBM, USA). The ROM, grip strength, VAS for pain, and subjective functional scores were treated as measurement data and expressed as mean ± standard deviation. Paired *t*‐tests were used to compare preoperative and follow‐up results. A *p*‐value of < 0.05 was considered statistically significant.

## 3 Results

The average operative time was 3.4 ± 1.4 h. The long‐stem prosthesis required more operative time compared to the plate osteosynthesis design (4.5 ± 0.7 h vs. 2.3 ± 0.4 h). The average blood loss was 60.0 ± 29.4 mL.

**FIGURE 4:**
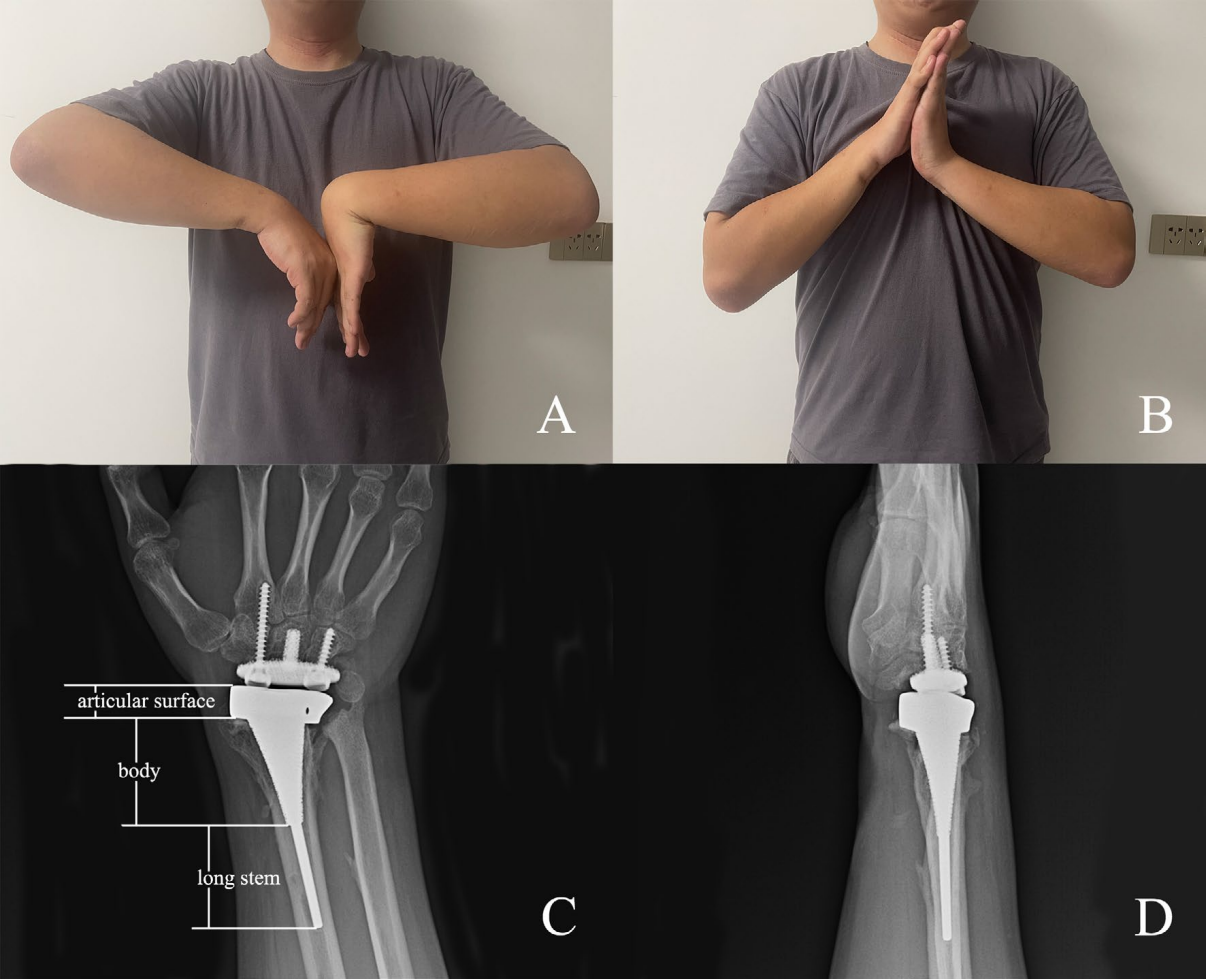
Postoperative range of motion and radiography of Patient 2 after 18 months. (A, B) Range of wrist flexion and extension. (C, D) The radiographic appearance of the prosthesis at the final follow‐up.

**FIGURE 5:**
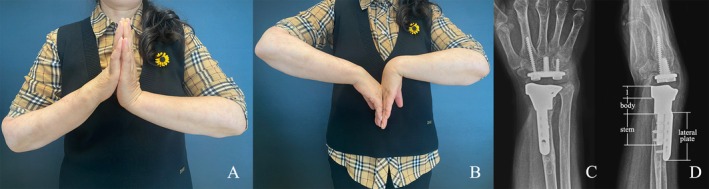
Postoperative range of motion and radiography of Patient 4 after 6 months. (A, B) Range of wrist flexion and extension. (C, D) The radiographic appearance of the prosthesis at the final follow‐up (D1) The articular surface structure.

The follow‐up period ranged from 6 to 18 months, with an average of 11.9 ± 6.7 months. Functional outcomes at the final follow‐up showed an ROM as follows: flexion 27.0° ± 18.6°, extension 23.3° ± 5.7°, radial deviation 6.0° ± 6.9°, ulnar deviation 19.8° ± 6.0°, pronation 52.5° ± 29.6°, and supination 49.3° ± 18.7° (Table 1, Figures 4A,B and 5A,B). The average flexion‐extension arc increased from 20.8° preoperatively to 50.3° postoperatively, reflecting a 142.2% significant improvement (*p* < 0.05). Improvements in other ranges of motion, grip strength, functional scores, and pain, as measured by the VAS, are shown in Table 2. A significant difference was observed between pre‐operative and final follow‐up values for quick DASH, PRWE, and the arc of radial‐ulnar deviation and supination‐pronation.

**TABLE 1:** Clinical evaluation results of the patients.

Patient	Original disease	Prosthesis type	Follow‐up (month)	Flexion ‐extension arc (°)	Grip strength (kg)	PRWE^b^	quickDASH^c^	Mayo	VAS of pain^d^
1	GCT^a^	Long‐stem	16	30	21	29	18	60	1
2	Trauma	Long‐stem	18	60	10	23	11	75	2
3	Severed wrist	Plate	6	51	5	51	68	40	3
4	GCT^a^	Plate	6	60	4	23	16	65	0

^a^ GCT: giant cell tumor.

^b^ PRWE: Patient‐Rated Wrist Evaluation.

^c^ quickDASH: Quick Disabilities of the Arm, Shoulder, and Hand.

^d^ VAS: visual analog scale of pain.

**TABLE 2:** Comparisons of evaluations between presurgery and final follow‐up.

	Flexion ‐extension arc (°)	Radial – ulnar deviation arc (°)	Supination– pronation arc (°)	Grip strength (kg)	PRWE	quickDASH	Mayo	VAS of pain
Before	20.8 ± 13.3	8.8 ± 11.7	39.3 ± 53.3	5.5 ± 7.2	69.3 ± 18.6	75.7 ± 7.2	27.5 ± 18.5	3.8 ± 1.5
After	50.3 ± 14.2	25.8 ± 4.0	101.8 ± 44.4	10.0 ± 7.8	31.5 ± 13.3	28.3 ± 26.7	60.0 ± 14.7	1.5 ± 1.3
*T* value	3.77	3.88	3.49	3.12	−4.13	−4.51	2.71	−1.71
*p*	0.033	0.030	0.040	0.053	0.026	0.020	0.073	0.19

The clinical outcomes at 6 weeks, 3 months, 6 months, and 1 year are summarized in Table 3. Most outcomes improved over time, except for the ROM, which showed a slight decrease at the 1‐year follow‐up.

**TABLE 3:** Clinical evaluation result in different time points.

	Preoperation	6‐week	3‐month	6‐month	1‐year^a^
Flexion ‐extension arc (°)	20.8 ± 13.3	39.5 ± 22.5	47.3 ± 9.6	48.5 ± 9.9	46.0 ± 14.1
*p* *		0.036	0.001	0.029	
Grip strength (kg)	5.5 ± 7.2	7.0 ± 5.7	9.0 ± 6.6	9.8 ± 7.3	15.0 ± 7.1
*p* *		0.196	0.066	0.030	
PRWE	69.3 ± 18.6	44.8 ± 19.3	43.0 ± 21.6	33.3 ± 13.0	28.0 ± 5.7
*p* *		0.195	0.051	0.021	
Quick DASH	75.7 ± 7.2	41.3 ± 21.6	38.0 ± 24.3	35.0 ± 22.9	22.5 ± 0.7
*p* *		0.094	0.019	0.010	
Mayo	27.5 ± 18.5	43.8 ± 17.0	46.3 ± 16.5	53.8 ± 11.1	57.5 ± 10.6
*p* *		0.290	0.127	0.060	
VAS of pain	3.8 ± 1.5	1.0 ± 1.4	1.5 ± 1.3	1.5 ± 1.3	1.5 ± 0.7
*p* *		0.092	0.093	0.093	

^a^ Using data from the two patients with follow‐up exceeding 1 year.

* Paired *t*‐tests were performed to compare preoperative and follow‐up results.

When asked about their expectations for the prosthesis, one patient chose “not worse than now” and three chose “improvement.” When asked if they would choose the procedure again, all patients responded “definitely would.” No complications such as loosening, infection, rupture, or prosthesis dislocation were observed (Figures 4C,D and 5C,D). Additionally, there were no recurrences of the bone or metastases in tumor patients.

## 4 Discussion

The main finding of this study was that the bespoke 3D‐printed metal prostheses led to significant improvements in clinical outcomes for patients with severely damaged wrists.

### 4.1 Design of the Prosthesis

Salvaging a severely damaged joint has consistently been a challenge in orthopedics. Whether caused by trauma, tumors, inflammation, failed reconstructions, or periprosthetic fractures, these cases typically share common characteristics: extensive damage to the articular surfaces, distal radius instability, and osteoporosis in the remaining bone. Strengthening the stability is an essential choice in prosthesis design for these cases.

The long stem prostheses in our study demonstrated excellent pain relief and functional satisfaction. No complications, such as loosening or signs of dislocation, have been found so far. However, Long‐term observation is necessary. The long‐stem design is a well‐established approach for achieving more robust fixation in the knee, hip, and shoulder [12, 13, 14]. Although some evidence suggests that long stems increase stress transfer while shielding the proximal bone [18], the compromised bone environment in wrists like those in our study does not support the use of short stems. Additionally, recent studies comparing long and short stem prostheses for the knee have found no significant difference between the two [19]. In hip prostheses, long femoral stems have demonstrated lower complication rates and similar functional outcomes compared to standard stems [20]. Revisions for periprosthetic femoral fractures using long‐stem prostheses enabled early, pain‐free weight‐bearing without compromising fracture healing in elderly patients with osteoporotic bone [21].

The plate osteosynthesis design in our study was an innovation for wrists requiring extensive bone resection, and it was applied later than the long stem type. The clinical outcome was acceptable, and patient satisfaction was high, possibly related to the low preoperative function lowering patient expectations. The combination of prosthesis and plate represents a newer design in hip and knee implants. The implementation varies based on the condition. In some hip prosthesis designs, a plate is added parallel to the intramedullary stem and connected with screws, yielding satisfactory clinical outcomes [12, 17]. This design enables immediate full weight‐bearing after total hip arthroplasty, which may account for the superior functional scores compared to long stem designs [12].

For the distal component, no major design changes have been made compared to the company's previous normal TWA prosthesis. Although loosening primarily occurs in the distal component of the total wrist prosthesis, the rate of this complication has been reduced to an acceptable level through the use of press‐fit fixation and the shortening of the screws and fixation rod [22].

### 4.2 Comparison With Arthrodesis

Traditional treatment for severely destroyed wrists typically involves wrist arthrodesis, which generally yields acceptable functional outcomes. Our results demonstrated a slight advantage compared to some of the reported outcomes of this procedure. For instance, Reigstad's 2019 study, involving 63 wrists with an average follow‐up of 11 years, reported quickDASH and PRWE scores of 36 and 40, respectively [23]. Similarly, in 2023, Roulet et al. reported quickDASH and PRWE scores of 30.3 and 32.5 with a 5‐year follow‐up [24]. However, more favorable results for arthrodesis have also been reported. For example, in Wagner's 14‐year follow‐up study, quickDASH and PRWE scores were 29 and 21, respectively [25].

### 4.3 Comparison to Other Prosthetic Designs

All patients in our study underwent multiple surgeries, with three patients undergoing their third procedure. As a result, their soft tissue conditions were significantly worse compared to those in most previous studies on arthroplasty. Despite this, our ROM and Mayo score are comparable to those reported in previous joint replacement studies (Table 4) [2, 6, 7, 8, 10, 11, 26, 27, 28, 29, 30, 31, 32]. Furthermore, the QuickDASH score performed even better, falling within the relatively good range established by these studies. The PRWE result was also acceptable. The patients were profoundly satisfied with the results, partly due to the conclusion of their long and arduous journey toward achieving acceptable hand function. An interesting case was Patient 2, who presented with a severe wrist deformity, nearly fixed at 30° of ulnar deviation and 45° of flexion prior to the procedure. Despite ongoing limitations in radial deviation after surgery, the patient reported high satisfaction with the replacement, attributing it to the positive impact on his work.

**TABLE 4:** Results from previous studies on wrist arthroplasty.

Research	Prostheses	*n*	Follow‐up (month)	FE‐arc (°)	Function		VAS	Grip (kg or %)^b^	Complication		Survival
					Scale	Score			Rate (%)	Symptom	
TWA for patients without bone defects											
2021‐Martínez	Universal 2	22	78	64	qDASH^c^	26	1	16	9	Aseptic implant failure (2)	91% (6.5 years)
					Mayo	61					
2013‐Boeckstyns	RE‐motion	65	72	60	qDASH	42	3	15	8	Loosening (4), stiffness (1)	90% (9 years)
2019‐Honecker	RE‐motion	23	120	83	qDASH	38	2.8	14	35	Infection (1), loosening (3), CTS^a^ (2), EPL rupture (1)	69% (10 years)
2013‐Nydick	Maestro	7	56	80	PRWE	31	2	65%	14	Fixed contracture (1)	NA^d^
2022‐Ota	DARTS	18	6	59	NA	NA	NA	NA	0	NA	NA^d^
PWA for patients with bone defects											
2009‐Natarajan	Custom‐made	24	78	45	MSTS	74%	NA	NA	17	Skin necrosis (2), infection (2), loosening (2)	88% (10 years)
2015‐Zhang	Custom‐made	11	55	70	MSTS	80%	NA	71%	9	Superficial infection (1)	NA^d^
2016‐Wang	Custom‐made	10	36	42	Mayo	68	2	68%	30	Loosening (1), subluxation (2)	NA^d^
2018‐Lu	Custom‐made	11	14	91	DASH	19	2.3	NA	0	NA	NA^d^
2020‐Wang	Custom‐made	15	31	106	Mayo	71	1.3	64%	40	Subluxation (3), DRUJ separation (3)	67% (5 years)
TWA for patients with bone defects											
2013‐Damert	Argomedical	1	24	55	DASH	25	4	16 (35%)	0	NA	NA^d^
2020‐Damert	Argomedical	1	120	NA	DASH	27	NA	18	100	Sintering of the radial component	NA^d^
2013‐Hariri	Argomedical	1	33	90	qDASH	53	2	17	100	DRUJ subluxation	NA^d^
Our result	Custom‐made	4	12	50	qDASH	28	1.5	10	0	NA	NA^d^
					PRWE	32					
					Mayo	60					

^a^ CTS: Carpal tunnel syndrome.

^b^ Kg or percentage compared to the contralateral side.

^c^ qDASH: QuickDASH.

^d^ These studies did not report the Kaplan–Meier survival rate but indicated no prosthetic failure.

Regarding TWA performed as a primary procedure, numerous studies have been published. For treating pancarpal posttraumatic arthritis, Nydick et al. reported DASH scores of 38 and 29 and PRWE scores of 73 and 31 for the arthrodesis and arthroplasty groups, respectively [28]. Martínez Villén's 2021 study reported quickDASH and PRWE scores of 26 and 26 for the Universal 2 prosthesis, which has demonstrated survival rates of 81%–91% in prior research [2, 33, 34, 35, 36]. Other fourth‐generation prostheses, such as RE‐MOTION and Maestro, have shown survival rates ranging from 69% to 95.7% [26, 27, 33, 37] and 93% to 100% [33, 38, 39], respectively.

There are limited studies reporting salvage procedures with prostheses. The primary challenge in this condition is managing the severely deformed structures or filling the large bone defects left after removing the affected area. Wrist replacement of large bone defects has been more commonly seen after bone tumor resection, with partial wrist arthroplasty (PWA) being the most commonly used method. Considering that bone defects following tumor resection predominantly occur on the radius, similar to many cases of reconstruction failure, their experience is also worthy of consideration. Early reports on PWA predominantly featured standardized designs, but in recent years, customized prostheses have increased. Currently, bespoke PWA appears to offer improved ROM for patients with large bone defects, as evidenced by reported flexion‐extension arcs ranging from 42° to 106° [6, 7, 8, 30, 31, 40] and favorable functional scores, with MSTS (Musculoskeletal Tumor Society) scores ranging from 74% to 83% [6, 8, 30, 40] and Mayo scores from 68 to 71 [8, 31].

Reports on TWA for treating large bone defects are scarce. Damert et al. published a case report on a customized prosthesis, based on the Universal 2, for the treatment of distal radius GCT. At the 2‐year follow‐up, the prosthesis demonstrated a flexion‐extension arc of 55° and favorable functional results with a DASH score of 25 [32]. The satisfying result continued to the 10‐year follow‐up [10]. Hariri et al. also reported on a customized prosthesis based on the RE‐Motion design, yielding a flexion‐extension arc of 90° and a quickDASH score of 52.6 at the 33‐month follow‐up [11]. Both prostheses were from the Swiss company Argomedical. A polyethylene spacer around the stem of the proximal component was the main customized part to fill bone defects. However, the procedures described in these studies are all primary surgeries performed immediately after the en‐bloc procedure, during which the soft tissue condition is relatively favorable.

### 4.4 Management of the Distal Radioulnar Joint (DRUJ)

Among the four patients, three displayed significant limitations in supination and pronation preoperatively. For these patients, a wafer procedure was performed during surgery, except for Patient 3, whose ulnar head had been resected in the previous replantation operation. Postoperatively, all of these patients experienced improvements in pronation–supination ROM. For the patient who did not undergo the wafer procedure (Patient 4), her postoperative supination ROM also had a mild improvement from 43° to 65°. During the follow‐up, she reported occasional mild ulnar‐side pain when reaching the limit of supination; however, the discomfort was minimal and did not affect her daily life. Patients who underwent the wafer procedure did not report any discomfort during rotation.

### 4.5 Limitations and Prospect

We recognize several significant limitations in our findings: The sample size was limited, partly due to the relatively low incidence of the condition and also the patients' willingness to restore the ROM of wrist. For those patients willing to undergo arthrodesis, this option was recommended first. Furthermore, the follow‐up period for this prosthesis was relatively brief. As a result, the long‐term performance and potential complications have not yet been fully assessed, highlighting the need for continued observation. Although no complications have been observed so far, we are well aware of the potential for complications in the future, such as loosening, peri‐prosthetic fracture, and instability of the distal radioulnar joint. In terms of prosthesis design, long‐stem prostheses exhibit a stronger stress shielding effect compared to short‐stem prostheses, which in turn increases the risk of osteolysis [41]. During subsequent follow‐ups, we will emphasize radiological evaluations of the prosthesis to assess whether signs of periprosthetic bone resorption are present. Additionally, the postoperative ROM in this study is relatively limited, largely due to the patient's soft tissue conditions. In the future, we aim to achieve better functional outcomes by optimizing the match between activity demands and the prosthesis design's ROM. Overall, we believe that TWA using a customized mental 3D printed prosthesis could be considered a viable therapeutic option for patients with extensive destruction of the wrist joint.

Larger studies with extended follow‐up periods are necessary to validate the long‐term efficacy of this surgery. We will carefully select suitable candidates and continue to explore the application of total wrist joint prostheses in the field of severely destroyed wrists. Additionally, we will continue to monitor the functional outcomes and prognosis of postoperative patients. In terms of prosthesis design, we will explore reducing the use of long stems to minimize the stress shielding effect. At the same time, we will further optimize the prosthesis's joint surface design to align with the functional motion of the wrist. This will enhance the efficiency of the designed ROM, ultimately improving postoperative functional outcomes for patients.

## 5 Conclusion

In the four patients with severely destroyed wrist joints, customized 3D‐printed total wrist joint prostheses achieved acceptable mobility and functionality, as well as high patient satisfaction. No complications were observed during follow‐up. The use of 3D‐printed total wrist prostheses can be considered a viable therapeutic option for treating heavily damaged wrists for various reasons.

## Author Contributions

**Shanlin Chen:** conceptualization and project administration. **Qipei Wei:**. investigation and writing (original draft), formal analysis. **Min Liu:** resources. **Chang Liu and Shanlin Chen:** supervision and writing (review and editing).

## Conflicts of Interest

The authors declare no conflicts of interest, except for Min Liu, who is employed by AK Medical, Beijing, China. This company provided the prosthetic devices used in the research. The author's affiliation does not influence the study's design, data collection, analysis, or interpretation.
